# Dietary networks identified by Gaussian graphical model and general and abdominal obesity in adults

**DOI:** 10.1186/s12937-021-00746-w

**Published:** 2021-10-27

**Authors:** Ahmad Jayedi, Nasim Janbozorgi, Kurosh Djafarian, Mir Saeed Yekaninejad, Sakineh Shab-Bidar

**Affiliations:** 1 Food Safety Research Center (salt), Semnan University of Medical Sciences, Semnan, Iran; 2 Department of Community Nutrition, School of Nutritional Science and Dietetics, Tehran University of Medical Sciences, P. O. Box 14155/6117, Tehran, Iran; 3 Department of Clinical Nutrition, School of Nutritional Science and Dietetics, Tehran University of Medical Sciences, Tehran, Iran; 4 Department of Epidemiology and Biostatistics, School of Public Health, Tehran University of Medical Sciences, Tehran, Iran

**Keywords:** Abdominal obesity, Dietary network, Dietary pattern, Diet quality, Obesity

## Abstract

**Background:**

Gaussian graphical model (GGM) has been introduced as a new approach to identify patterns of dietary intake. We aimed to investigate the link between dietary networks derived through GGM and obesity in Iranian adults.

**Method:**

A cross-sectional study was conducted on 850 men and women (age range: 20–59 years) who attended the local health centers in Tehran. Dietary intake was evaluated by using a validated food frequency questionnaire. GGM was applied to identify dietary networks. The odds ratios (ORs) and 95% confidence intervals (CIs) of general and abdominal adiposity across tertiles of dietary network scores were estimated using logistic regression analysis controlling for age, sex, physical activity, smoking status, marital status, education, energy intake and menopausal status.

**Results:**

GGM identified three dietary networks, where 30 foods were grouped into six communities. The identified networks were healthy, unhealthy and saturated fats networks, wherein cooked vegetables, processed meat and butter were, respectively, central to the networks. Being in the top tertile of saturated fats network score was associated with a higher likelihood of central obesity by waist-to-hip ratio (OR: 1.56, 95%CI: 1.08, 2.25; *P* for trend: 0.01). There was also a marginally significant positive association between higher unhealthy network score and odds of central obesity by waist circumference (OR: 1.37, 95%CI: 0.94, 2.37; *P* for trend: 0.09). Healthy network was not associated with central adiposity. There was no association between dietary network scores and general obesity.

**Conclusions:**

Unhealthy and saturated fat dietary networks were associated with abdominal adiposity in adults. GGM-derived dietary networks represent dietary patterns and can be used to investigate diet-disease associations.

## Background

There was a rising trend in obesity during the past decades globally [1]. Currently, approximately one-third of the world’s population is overweight or obese [2]. Adiposity, mainly reflected by increased body mass index (BMI), is linked to a greater risk of cardiometabolic disease and mortality [3–6] and thereby has imposed a substantial financial burden either in developed or in developing countries [7]. Traditionally, obesity has been thought to be a consequence of positive energy balance [8]. However, there is evidence that intake of some food groups, independent of total energy intake, may be associated with the risk of adiposity [9, 10].

In general, food groups are consumed in different combinations called dietary patterns. Dietary patterns are combinations of foods or food groups that are different from dietary behaviours, which are related to behaviors such as skipping meals, snacking, drinking sweetened beverages, and eating fast food. The major identified data-driven dietary patterns are Western-style dietary pattern, rich in red and processed meat, refined grains, and high fat dairy, as well as Prudent or healthy dietary pattern rich in fruit and vegetables, whole grains, fish, and low fat dairy [11, 12].

It is proposed that dietary patterns represent a broader perspective of food and nutrient consumption, and may thus be more predictive of disease risk than individual foods or nutrients [13]. Dietary pattern analyses are increasingly used to investigate diet-disease associations [14]. It has been shown that higher adherence to a healthy diet may be associated with a lower likelihood of adiposity and in contrast, adopting a Western-style dietary pattern may promote adiposity [15–20].

Data-reduction statistical methods such as principal component (PCA) [21] or cluster analysis [22] and reduced rank regression [23] are useful techniques that have been frequently used to characterize patterns of dietary intake considering potential inter-relations between food groups [24]. A recent umbrella review indicated that PCA is the most common statistical approach to characterize patterns of dietary intake [14], because it considers correlation or covariance existed between food groups to create uncorrelated linear combinations entitled components or patterns [25].

However, PCA and other common statistical methods do not demonstrate pairwise correlation between food groups. Gaussian graphical model (GGM) is recently used as an innovative approach exploring patterns of dietary intake [26]. This graphical method shows the pairwise correlation between food groups, independent of the effects of other food groups [27], and thereby can show that how food groups are consumed in relation to one another [26]. The conditional independence analysis constructs the networks of foods that shows the underlying structure of the dataset. This method presents a graphical perspective from the link between food groups and identifies dietary networks representing patterns of dietary intake [28].

GGM is a novel statistical approach to explore patterns of dietary intake. However, patterns identified through this method have not been investigated in relation to the risk of adiposity. In addition, Iranian eating habits are changing rapidly towards a Western-style eating pattern [29]. GGM can show food groups that are central to the potentially healthy and unhealthy dietary networks and thus, can help determine what food groups are the main constructors of dietary patterns. This information cannot be obtained by other data-driven approaches and can help developing more efficient dietary guidance to prevent rising trend in adherence to Western dietary patterns in Iran. In this study, we, therefore, aimed to describe dietary networks identified by GGM, representing patterns of dietary intake in a sample of Iranian adults and to investigate the potential association of these dietary patterns with general and abdominal adiposity.

## Materials and methods

### Study participants

The present cross-sectional study was performed in Tehran, capital of Iran, from 2018 to 2019. The formula used for sample size calculation was as follows: *n* = (pqz2)/E2 [30, 31]. Considering the prevalence of overweight and obesity in Tehranian adults (65%) [32], an error coefficient of d = 0.04 and at α level of 0.05, the sample size of 546 participants was obtained. With a design effect of 1.5 and to compensate for the potential exclusion of participants due to under- and over-reporting of total energy intake, or attrition due to other reasons, the final sample size of 850 participants was selected for inclusion.

For recruitment of participants, the following criteria were applied: apparently healthy men and women, aged 20 to 59 years who attended the local health care centers during the study period and had the willingness to take part in the study. Apparently healthy was defined as adults without existing non-communicable chronic diseases including cardiovascular disease, type 2 diabetes, cancers, and respiratory, renal, and autoimmune disorders.

A two-stage cluster sampling was used for the recruitment of participants from healthcare centers. First, a list of all healthcare centers that existed in five districts of the city (North, South, East, West and center) was provided. Then we randomly chose eight health centers from each district (40 health centers). Finally, to obtain the number of participants in each health center, we divided the total sample size (850) by the number of health centers (40).

### Ethical approval

The ethical committee of the Tehran University of Medical Sciences approved the study protocol and informed consent form (Ethic Number: IR.TUMS.VCR.REC.1397.157). All patients received written information regarding the background and procedures of the study and gave written informed consent before entering the study.

### Data collection

Through a face-to-face interview, participant’s demographic characteristics were obtained by using pre-specified data extraction forms. A trained interviewer completed a questionnaire designed to assess the participants’ demographics including age (year), gender, educational level (illiterate, under diploma, diploma, educated), marital status (married or other [not married or divorced]), occupation (employed, retired, house-keeper, or unemployed), and smoking status (never smoked, former smoker, current smoker).

### Dietary assessment

Dietary intake was assessed by using a reliable and validated 168-item food frequency questionnaire [33]. During a private face-to-face interview and by a trained dietitian, the frequency (daily, weekly, monthly, and yearly) and amount of each food item during the past year was recorded. Dietary intakes were then converted to g/d according to household measures [34]. Intake of energy and nutrient content of foods was estimated by using Nutritionist IV software based on the US Department of Agriculture food composition database modified for Iranian foods [35].

### Physical examinations

Weight was measured using a digital adult scale (Seca model 808, measurement accuracy +/− 100 g) [36]. Participant’s height was measured unshod using a wall stadiometer with a precision of 1 cm (Seca, Germany) [36]. BMI was calculated as weight divided by the square of height (kg/m^2^). Waist circumference was measured with a tape measure to the nearest 0.1 cm between the iliac crest and the lowest rib during exhalation. Hip circumference was recorded below the iliac crest, by measuring the maximum circumference around the buttocks and then, waist-to-hip ratio (WHR) was calculated.

Physical activity was assessed by using the International Physical Activity Questionnaire [37] and was recorded as metabolic equivalent minutes per week (MET-min/week) [38]. Participants were grouped into two categories including “no or low physical activity” (< 3000 MET-min/week) and “moderate or high physical activity” (> 3000 MET-min/week).

### Definition of general and abdominal adiposity

General adiposity was defined as BMI ≥ 30 kg/m^2^ [39]. Central adiposity was defined as follow: waist circumference greater than 102 cm for men and 88 cm for women [40], and WHR greater than 0.90 for men and 0.85 for women [41].

### Statistical analysis

GGM was used to explore networks of dietary intake of participants. These types of statistical analyses are a class of methods that are increasingly used for exploratory analysis [42]. GGMs are graphical models that show the conditional independence structure in the data set by assessing the pairwise correlation between two variables controlling for others. GGMs assume a multivariate normal distribution for underlying data and can infer a direct relation between variables in a given data set without prior knowledge [43]. The use of GGMs for exploring conditional independence structures between food intake variables is an emerging and promising approach.

For the purpose of the present study, dietary intakes of participants were classified into 35 food groups (Table 1). The analysis of GGM was performed in R (version 3.4.3, R) [44]. A sparse inverse covariance (precision) matrix was estimated from the data using graphical lasso (least absolute shrinkage and selection operator) in R package “glasso” [26]. Communities, sets of closely related links, were detected within all identified networks to facilitate interpretation using the R package “linkcomm”, which can detect nested and overlapping communities in networks [45].

**Table 1:** List of food groups included in the analysis of GGM to derive dietary networks

n	Food groups	n	Food groups
1	Red meat	19	Grains
2	Processed meat	20	Nuts
3	Organ meat	21	Legumes
4	Fish	22	Snacks
5	Low-fat diary	23	Salty snacks
6	High-fat dairy	24	Cookies/cake
7	Fresh fruit	25	Pickles and flavors
8	Dried fruit	26	Cheeps/puff
9	Canned fruit	27	Sugar sweetened beverages (drinks)
10	Fruit juices (100%)	28	Sauces
11	Raw vegetables	29	Butter
12	Cooked vegetables	30	Margarine
13	Cabbages	31	Animal fat
14	Garlic	32	Vegetable oils
15	Mushroom	33	Egg
16	Other vegetables	34	Tea
17	Potatos	35	Coffee
18	Side dish		

GGM-derived dietary networks consist of nudes and edges. Nudes indicate food or food groups. Edges show conditional dependencies between food groups indicated by partial correlation coefficients. The width of the edges indicates the strength of the correlations that existed between food groups. Partial correlations ≥ ± 0.20 were considered strong [46]. Continuous lines represent positive partial correlations and broken lines represent negative partial correlations. Communities were indicated by nodes and edges of the matching color. A combination of three or more nudes that were related to each other formed a dietary network. The absence of an edge between food groups indicates conditional independence considering all other variables [47]. Food groups that belonged to more than one community were evaluated for centrality to determine the potential importance of a food group based on the number of communities it belongs to [48].

To investigate the link between GGM-derived dietary networks and adiposity in the participants, all major networks were scored. For this purpose, dietary intake variables included in each network were standardized to the same mean (i.e., ‘0’) and 1 standard deviation. In the second step, standardized intakes of food groups were multiplied by their factor loading scores (positive or negative) obtained by PCA. Then, the score of food groups within each network was added together to calculate network scores. The network scores were then categorized in tertiles and the characteristics of participants across tertiles of dietary networks were compared using χ2 for categorical variables and ANOVA test for continuous variables. The odds ratios (ORs) and 95% confidence intervals (CIs) of general and abdominal adiposity across tertiles of network scores were estimated using logistic regression analysis controlling for age, sex, physical activity, smoking status, marital status, energy intake and menopausal status (for women). The analyses were performed using SPSS software, version 22 (SPSS Inc., Chicago, IL, USA). A two-sided *p*-value < 0.05 was considered significant.

## Results

The present cross-sectional study included 850 adults, of whom 69% were women. The general characteristics of the study participants are presented in Table 2. Participants were on mean 44.7 ± 10.8 years old and the mean BMI was 27.8 ± 5.6 kg/m^2^. There is no difference in terms of age and BMI across either sex. The mean energy intake was 2586 ± 1140 kcal/d. The majority of participants were nonsmoker (91%) and had a sedentary lifestyle (< 3000 MET-min/week) (63%).

**Table 2:** General characteristics of the study participants (*n* = 850)

Variable^**a**^	Total population, ***n*** = 850	Women, ***n*** = 584	Men, ***n*** = 266
**Age (years)**	44.7 ± 10.8	44.5 ± 11.1	45.2 ± 10.1
**Body weight (Kg)**	73.4 ± 13.5	70.2 ± 11.8	80.6 ± 14.3
**Height (cm)**	162.0 ± 8.9	159.0 ± 7.2	170.0 ± 7.2
**BMI (kg/m**^**2**^**)**	27.8 ± 5.6	27.9 ± 6.1	27.6 ± 4.1
**Waist circumference (cm)**	92.0 ± 12.4	90.5 ± 12.3	95.3 ± 12.0
**WHR (unit)**	0.88 ± 0.11	0.86 ± 0.13	0.90 ± 0.12
**Physical activity (% low)**	63%	65%	61%
**Education (% educated)**	34.3%	41.9%	38.0%
**Occupation (% employed)**	26%	19.3%	40.2%
**Smoking status (% current)**	5.2%	1.8%	12.4%
**Marital status (% married)**	80.9%	77.2%	89.1%
**Post-menopausal (%)**	–	27.8%	–
**Dietary intake**			
**Energy (kcal/d)**	2586 ± 1140	2745 ± 1120	2487 ± 1146
**Carbohydrate (g/d)**	354 ± 153	380 ± 155	343 ± 150
**Fat (g/d)**	82.3 ± 51.3	86.7 ± 50.1	80.2 ± 51.5
**Protein (g/d)**	86.4 ± 48.0	93.2 ± 39.7	83.3 ± 51.0

Abbreviations: *BMI* body mass index, *DBP* diastolic blood pressure, *SBP* systolic blood pressure, *WHR* waist-to-hip ratio

^a^Values are mean ± standard deviation for continuous variables and (%) for categorical variables

### Characteristics of dietary networks identified by GGM

**Fig. 1:**
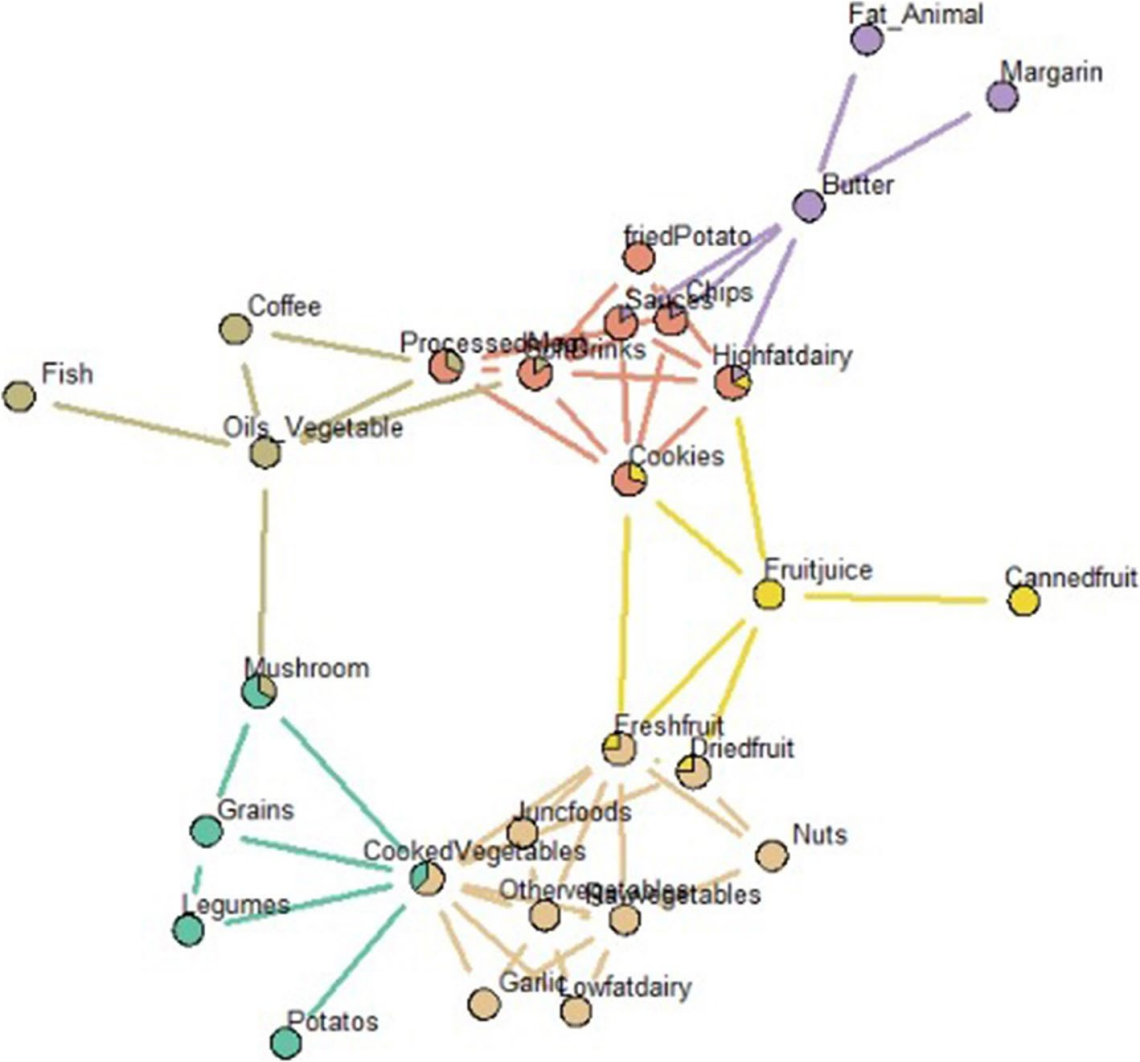
Dietary networks identified through Gaussian graphical model in Iranian adults (*n* = 850)

**Fig. 2:**
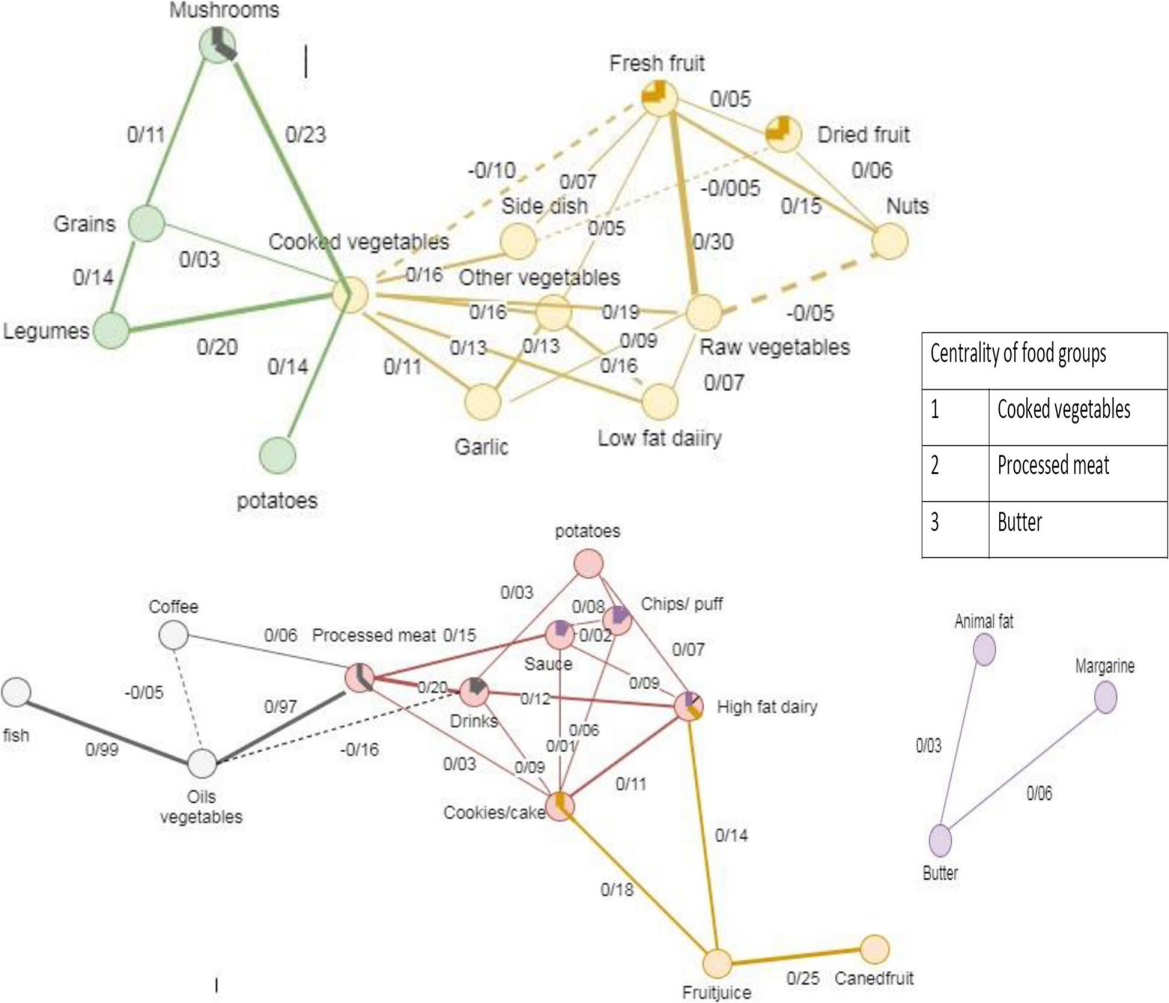
Detailed characteristics of three dietary networks identified by Gaussian graphical model. Nodes represent food groups. Edges represent conditional dependencies between food groups revealed by partial correlation coefficients. The absence of an edge between 2 food groups indicates conditional independence between them. Continuous edges show positive partial correlations while broken edges show negative partial correlations. Line thickness is proportional to the strength of the correlations between food groups. Communities are represented by matching node and edge colors. Centrality indicates importance of a food group based on the number of communities it belongs to

GGM analysis identified three major networks of dietary intake (Fig. 1), where 30 foods were grouped into six communities. The identified networks were healthy, unhealthy and saturated fats networks, wherein cooked vegetables, processed meat and butter were, respectively, central to the networks (Fig. 2). The central situation of the aforementioned food groups indicates their important position in the identified dietary networks.

The healthy dietary network is composed of two communities, where cooked vegetables was central to the network (Fig. 2). Starting in the upper left, the first community consisted of cooked vegetables, mushrooms, grains, legumes and baked potato, where almost all foods were partially correlated to each other. The network indicated that the intake of a baked potato was conditionally dependent on the intake of cooked vegetables (partial correlation = 0.14). The second community in the upper right was composed of nine food groups, among which there was a strong positive correlation between fresh fruit and raw vegetables (partial correlation = 0.30). There was also a slight negative correlation between raw vegetables and nuts (partial correlation = − 0.05), fresh fruit and cooked vegetables (partial correlation = − 0.10), and side dish and dried fruit (partial correlation = − 0.005).

Another network identified in the participants was an unhealthy network and was made of three communities (Fig. 2). The network showed a central role of processed meat. The first community in the lower-left composed of five foods, wherein there was a strong positive correlation between vegetable oils and fish and processed meat (partial correlations = 0.99 and 0.97, respectively). There was also a slight negative correlation between vegetable oils and coffee and soft drinks (partial correlations = − 0.05 and − 0.16, respectively). The network indicated that the intake of fish was conditionally dependent on the intake of vegetable oils. There were also two relatively overlapping communities in the unhealthy network. The central community consisted of processed meat, fried potatoes, soft drinks, sauces, cookies and cakes, chips, and high-fat dairy products, wherein food groups were positive albeit weakly correlated to each other. The third community was made of cookies and cakes, high-fat dairy, fruit juices and canned fruit. The network indicated that the intake of canned fruit was conditionally dependent on the intake of fruit juices.

The third network in the lower right consisted of butter, animal fat, and margarine that were linked by slight positive correlations (Fig. 2). The saturated fats network indicated that the intake of animal fat and margarine was conditionally dependent on the intake of butter (partial correlations = 0.03 and 0.06, respectively). Butter was also central to the network.

Characteristics of the study participants across tertiles of dietary networks are presented in Table 3. There is no difference in terms of age, sex, anthropometric measures, physical activity levels, education level, and other characteristics across tertiles of identified dietary networks.

**Table 3:** Characteristics of the study participants across tertiles of dietary networks

Variables^**a**^	Healthy network			***P*** for trend^**a**^	Unhealthy network			***P*** for trend^**a**^	Saturated fats network			***P*** for trend^**b**^
	T1	T2	T3		T1	T2	T3		T1	T2	T3	
**n**	284	283	283		284	283	283		283	284	283	
**Age (years)**	44.2 ± 10.6	44.6 ± 10.8	45.4 ± 10.9	0.37	45.1 ± 10.6	44.9 ± 10.9	44.3 ± 10.8	0.70	44.9 ± 10.8	44.7 ± 10.7	44.6 ± 10.8	0.92
**Sex (% women)**	68.9%	70.1%	68.2%	0.67	67.9%	68.1%	70.1%	0.07	70.0%	69.0%	69.5%	0.25
**Body weight (kg)**	73.7 ± 13.4	73.5 ± 13.4	73.8 ± 13.8	0.78	72.8 ± 13.6	72.5 ± 12.7	74.9 ± 14.3	0.34	73.5 ± 14.7	73.2 ± 12.9	72.9 ± 12.7	0.61
**BMI (kg/m**^**2**^**)**	27.8 ± 4.9	27.9 ± 6.9	28.7 ± 4.5	0.96	27.8 ± 5.0	28.3 ± 7.2	27.7 ± 4.8	0.23	28.1 ± 7.3	27.6 ± 4.3	27.7 ± 4.7	0.37
**Waist circumference (cm)**	91.6 ± 11.9	91.6 ± 12.6	92.8 ± 12.7	0.41	90.8 ± 12.1	92.8 ± 12.3	92.8 ± 13.0	0.12	91.4 ± 12.5	92.0 ± 11.6	92.7 ± 13.5	0.77
**WHR (unit)**	0.88 ± 0.08	0.89 ± 0.16	0.88 ± 0.08	0.27	0.88 ± 0.16	0.89 ± 0.08	0.88 ± 0.08	0.71	0.88 ± 0.16	0.88 ± 0.08	0.89 ± 0.08	0.46
**Physical activity (% low)**	62.0%	63.9%	64.1%	0.36	65.5%	64.9%	62.7%	0.46	62.7%	62.8%	64.5%	0.58
**Smoking status (% current)**	5.0%	4.2%	3.8%	0.03	4.9%	5.8%	5.1%	0.14	4.9%	4.6%	5.7%	0.35
**Education (% educated)**	33.5%	34.0%	34.2%	0.71	33.5%	32.9%	34.0%	0.27	33.0%	34.1%	35.2%	0.08
**Occupation (% employed)**	26%	25%	25%	0.53	27%	26%	26%	0.58	24%	25%	26%	0.20
**Marital status (% married)**	80%	81%	81%	0.48	80%	79%	79%	0.32	81%	81%	80%	0.90
**Dietary intake**												
**Energy intake (kcal/d)**	2512 ± 1115	2553 ± 1082	2512 ± 1186	0.29	2320 ± 1040	2362 ± 1598	2082 ± 1680	0.95	2521 ± 1245	2668 ± 1028	2509 ± 1092	0.07
**Carbohydrate (g/d)**	412 ± 158	370 ± 158	371 ± 193	0.46	367 ± 164	402 ± 156	384 ± 100	0.66	366 ± 154	420 ± 167	368 ± 184	0.19
**Fat (g/d)**	78.2 ± 60.4	86.6 ± 50.9	82.3 ± 41.1	0.18	79.4 ± 56.3	81.9 ± 48.2	85.7 ± 48.9	0.32	82.9 ± 59.4	85.2 ± 48.7	78.7 ± 44.4	0.19
**Protein (g/d)**	87.4 ± 62.9	86.2 ± 38.7	85.5 ± 38.4	0.89	87.2 ± 62.9	88.3 ± 57.9	85.5 ± 38.4	0.89	85.1 ± 37.9	90.4 ± 67.8	84.3 ± 33.4	0.21

Abbreviations: *BMI* body mass index, *WHR* waist-to-hip ratio

^a^Values are mean ± standard deviation for continuous variables and (%) for categorical variables

^b^Obtained by ANOVA for continuous variables and χ2 for categorical variables

Table 4 shows the association between the GGM-derived dietary networks and likelihood of general and abdominal adiposity in the study participants. Higher adherence to GGM-derived healthy, unhealthy, and saturated fats networks was not associated with likelihood of general adiposity. Being in the second (OR: 1.43, 95%CI: 0.99, 2.09) or third (OR: 1.37, 95%CI: 0.94, 2.01) tertiles of unhealthy dietary network score was not related to the odds of having abdominal obesity, defined by increased waist circumference (*P* for trend = 0.09). In addition, being the third tertile of GGM-derived saturated fats network score was associated with higher odds of abdominal adiposity as assessed by WHR, either in the crude (OR: 1.61, 95%CI: 1.16, 2.25; *P* for trend = 0.004) or in the maximally adjusted model (OR: 1.56, 95%CI: 1.08, 2.25; *P* for trend = 0.01).

**Table 4:** The association between dietary networks and general and central adiposity^a^

Dietary networks	Tertile 1	Tertile 2	Tertile 3	P for trend^**b**^
**BMI ≥ 30 kg/m**^**2**^				
**Healthy network**				
Crude	1.0	0.98 (0.68, 1.41)	0.98 (0.68, 0.41)	0.92
Adjusted	1.0	1.08 (0.74, 1.58)	1.01 (0.69, 1.46)	0.95
**Unhealthy network**				
Crude	1.0	0.82 (0.57, 1.19)	0.94 (0.66, 1.35)	0.76
Adjusted	1.0	0.79 (0.54, 1.15)	0.99 (0.68, 1.44)	0.96
**Saturated fats network**				
Crude	1.0	0.98 (0.68, 1.42)	1.01 (0.70, 1.45)	0.92
Adjusted	1.0	0.91 (0.62, 1.34)	0.99 (0.68, 1.44)	0.98
**WC > 102 cm in men and 88 cm in women**				
**Healthy network**				
Crude	1.0	0.84 (0.60, 1.17)	1.20 (0.86, 1.67)	0.27
Adjusted	1.0	0.95 (0.63, 1.32)	1.30 (0.89, 1.89)	0.14
**Unhealthy network**				
Crude	1.0	1.36 (0.98, 1.90)	1.14 (0.82, 1.59)	0.42
Adjusted	1.0	**1.43 (0.99, 2.09)**	**1.37 (0.94, 2.01)**	**0.09**
**Saturated fats network**				
Crude	1.0	1.02 (0.73, 1.42)	1.15 (0.82, 1.59)	0.40
Adjusted	1.0	0.92 (0.63, 1.34)	1.20 (0.83, 1.75)	0.29
**WHR > 0.90 for men and 0.85 for women**				
**Healthy network**				
Crude	1.0	1.23 (0.88, 1.72)	1.16 (0.86, 1.67)	0.27
Adjusted	1.0	1.34 (0.93, 1.93)	1.18 (0.82, 1.70)	0.34
**Unhealthy network**				
Crude	1.0	1.25 (0.90, 1.74)	1.12 (0.81, 1.56)	0.47
Adjusted	1.0	1.19 (0.82, 1.70)	1.03 (0.71, 1.48)	0.85
**Saturated fats network**				
Crude	1.0	1.10 (0.79, 1.53)	**1.61 (1.16, 2.25)**	**0.004**
Adjusted	1.0	1.05 (0.73, 1.50)	**1.56 (1.08, 2.25)**	**0.01**

Abbreviations: *BMI* body mass index, *WC* waist circumference, *WHR* waist-to-hip ratio

^a^Data are expressed as odds ratio and 95% confidence interval

^b^Obtained by logistic regression analysis, controlling for age, sex, occupation, smoking status, education, marital status, menopausal status, physical activity, and energy intake

## Discussion

In the present cross-sectional study, we investigated the link between GGM-derived dietary networks and likelihood of general and abdominal adiposity in a sample of Iranian adults. The results suggested that being in the top tertile of saturated fat network score was strongly associated with the chance of having central adiposity as defined by WHR. There was also a non-significant positive association between higher adherence to the unhealthy dietary network and central adiposity as assessed by waist circumference. There was no association between unhealthy and saturated fats networks and the likelihood of general adiposity. Higher adherence to the healthy dietary network was not related to general and central obesity.

Our results regarding the association of unhealthy and saturated fats dietary networks and central obesity are consistent with those of previous studies. Previous research showed a similar association between unhealthy dietary patterns, identified by other statistical approaches such as PCA or factor analysis, and central obesity. A cross-sectional study in Iranian female teachers in Tehran indicated that higher adherence to a factor analysis-derived Western-style dietary pattern rich in red and processed meat, fried potato, butter, high-fat dairy, refined grains, sugar-sweetened beverages and pizza was associated with higher odds of central obesity [49]. The GGM-identified unhealthy network in our study was composed of several components similar to the Western-style dietary pattern reported by Esmaillzadeh et al. [49]. Both studies were conducted on Tehranian adults. However, our unhealthy pattern also included other foods as well, such as sauce, chips, coffee, vegetable oil and canned fruit.

A cross-sectional study in Canada applied factor analysis to derive patterns of dietary intake. The analyses indicated that a Western dietary pattern, defined by higher consumption of French fries, condiments, red and processed meats, refined grains, and regular soft drinks, was associated with larger waist circumference and WHR [50]. Heidemann et al. reported that higher adherence to factor analysis-derived processed food pattern, rich in high-sugar beverages, sweets and cakes, snacks, refined grains, potatoes, red and processed meat, eggs, beer, and butter was positively associated with abdominal adiposity within a nationally representative sample of 4000 German adults [51].

The Thai National Health Examination Survey IV indicated that factor analysis-derived meat pattern, characterized by a high intake of red and processed meat and fried foods, was associated with abdominal obesity in men, but not in women [52]. A baseline evaluation within a cohort study in the Mexican adults’ population indicated that a high protein, high-fat diet, characterized by a high factor loading score of red and processed meat, poultry, egg, and butter was positively associated with abdominal adiposity [53]. However, there is inconsistent evidence. A cross-sectional investigation in Lebanese adults in the Middle East failed to show an association between unhealthy dietary patterns, derived by factor analysis, and central obesity [54]. Other cross-sectional studies have reported similar null associations [55–57]. The inconsistent findings across studies may be due to different components of identified dietary patterns, different tools used to assess dietary intake, and different adjustment models.

Our GGM-derived unhealthy network shared similarities with components of unhealthy patterns identified in the aforementioned studies. In particular, two population-based investigations in Tehranian adults in Iran identified similar Western/unhealthy dietary patterns [49, 58]. In a population-based cross-sectional study in Tehranian female teachers, through factor analysis, authors identified a Western-type dietary pattern rich in red and processed meat, high-fat dairy, fried potato, refined grains, egg, sugar-sweetened beverages, and butter [49]. We also found a similar unhealthy network, with some additional components such as coffee, canned fruit, and sauce. In the Tehran Lipid and Glucose Study involving 1630 Tehranian adults, a PCA-derived high-fat, high sugar dietary pattern was identified, characterized by high intake of mayonnaise, butter, solid oil, sweet and salty snack, coffee, soda, high-fat dairy, and pizza [58], which was comparable with our GGM-identified saturated fats network.

Our GGM-derived healthy dietary network was also similar to factor analysis- and PCA-derived dietary patterns identified in other population-based studies in Tehran. Through factor analysis, a healthy dietary pattern rich in whole grains, fruit, vegetables, legumes, low-fat dairy, fish, and poultry was identified in Tehranian adults [49]. We also found other healthy food as well, such as nuts in our GGM-derived healthy network. In another population-based study in Tehran, a PCA-derived healthy dietary pattern, rich in fruit, vegetables, low-fat dairy, and dried fruit, was identified [58].

These results indicated that GGM can be used as a complementary approach to identify dietary networks reflecting patterns of dietary intake. A population-based cohort study in Germany indicated that GGM-derived dietary networks reflect dietary patterns and could be used to investigate diet-disease associations [28]. GGM describes internal patterns representing networks and indicates key interrelated food groups that may be potential candidates for further diet-disease investigations. In addition, GGM can show that how food groups are consumed in different combinations, which may be useful for interpreting the dietary patterns of the population. In contrast to traditional statistical approaches such as PCA or factor analysis, each food or food group can only be part of a specific dietary network at a time.

One of the main advantages of GGM is that it can identify food groups that are central to the networks. As presented above, Iranian eating habits are changing rapidly towards a Western-style eating pattern [29]. GGM can show food groups that are central to the potentially healthy and unhealthy dietary networks and thus, can help determine what food groups are the main constructors of the dietary patterns. For example, our analyses indicated that processed meat and butter are the two food groups that are central to the unhealthy dietary patterns in a sample of Tehranian adults. This information cannot be obtained by other data-driven approaches and can help developing more efficient dietary guidance to prevent the growing rise in Western dietary patterns in Iran.

Another advantage of GGM compared to traditional methods for exploring dietary patterns is links between food groups. Our saturated fat dietary network indicated correlations between butter, animal fat, and margarine, suggesting that these foods were consumed in relation to each other. These information can be used for identifying consumption probabilities of the foods identified in the network for each individual. Such probabilities would be helpful for modeling alternative intake patterns by modifying intake probabilities, which may help assess the impact of dietary behavior change or dietary recommendations.

In addition, our analyses revealed that processed meat consumption is central to the unhealthy dietary intake in the population studied in this research. GGM underscored its importance and presented the pattern of its consumption, i.e., how it is consumed in relation to other foods. The networks indicated positive correlations between processed meat and vegetable oils, drinks, high fat dairy, and sauce. This is interesting because the role of red meat for health outcomes is still a research agenda priority [59, 60].

There are some potential mechanisms to explain the associations observed in our study. The higher likelihood of central obesity observed in participants with high unhealthy and saturated fats network scores could be related to lower consumption of healthy foods and protective nutrients [17]. In addition, the saturated fat network is composed of dietary sources of saturated fats such as animal fats, butter, and margarine. The unhealthy network was also made of foods rich in saturated fats such as red and processed meats and high-fat dairy. There is evidence that high saturated fat intake through exerting unfavorable impacts on gut microbiota composition [61], inducing obesity-related gene expression [62], and reducing fat oxidation and daily energy expenditure [63], can accelerate adiposity. High saturated fat intake promotes insulin resistance and low-grade systemic inflammation, the two potential obesity-inducing pathophysiological mechanisms [63]. The 2008 report of the third FAO (Food and Agriculture Organization of the United Nations)/WHO (World Health Organization) Expert Consultation on Fats and Fatty Acids in Human Nutrition highlighted the need for shifting from fat quantity to quality [64]. The 2015 US Dietary Guidelines Advisory Committee (DGAC) report issued that it is more important to optimize types of dietary fat than reducing total fat [65].

The present cross-sectional study had several strengths. To our knowledge, this is the first population-based study to investigate the association between GGM-derived dietary networks, representing patterns of dietary intake, and general and abdominal adiposity in adults. Our results showed that GGM can be used as a supplementary approach for identifying eating patterns. We used trained dietitians and valid tools to obtain information from participants. In addition, we recruited a relatively large number of participants. Additionally, GGM minimizes the subjective choices when analyzing data for identifying dietary patterns and thereby, presents robust results.

There were also some important limitations for consideration. We did not identify meal-specific dietary networks and thus, future research can focus on the properties of meal-based dietary networks. We used a food frequency questionnaire for dietary assessment that has been shown to have some limitations in evaluating dietary information [66]. The cross-sectional design of our study is another limitation that highlights the need for prospective studies to confirm the findings. Finally, due to the lack of consumption of whole grains in the Iranian diet, we did not include whole grains in the GGM analysis.

## Conclusions

The present cross-sectional study describes GGM-derived dietary networks reflecting dietary patterns in a sample of Iranian adults. Our results indicated that being in the top tertile of saturated fat network scores was associated with a higher odds of abdominal adiposity. There was no association between dietary networks and general adiposity. GGM can show that how food groups are consumed in relation to one another and can determine food groups that are central to the dietary networks. This method shows the underlying structure of the dataset and thus, presents a graphical perspective from the link between food groups. This information could be used to present a better understanding of the construction of dietary patterns and can help to present more useful dietary guidance.

## Data Availability

The datasets used and/or analyzed during the current study are available from the corresponding author on reasonable request.

## Abbreviations

BMI: Body mass index
GGM: Gaussian graphical model.
PCA: Principal component analysis
WHR: Waist-to-hip ratio
